# An Overview of Challenges for the Future of Hydrogen

**DOI:** 10.3390/ma16206680

**Published:** 2023-10-13

**Authors:** Md Tanvir Ahad, Md Monjur Hossain Bhuiyan, Ahmed Nazmus Sakib, Alfredo Becerril Corral, Zahed Siddique

**Affiliations:** School of Aerospace and Mechanical Engineering, University of Oklahoma, Norman, OK 73019, USA; mhbhuiyan@ou.edu (M.M.H.B.); nazmus.sakib@ou.edu (A.N.S.); abecerril@ou.edu (A.B.C.);

**Keywords:** hydrogen embrittlement (HE), compatible materials, metals, polymers, diffusion coefficient

## Abstract

Hydrogen’s wide availability and versatile production methods establish it as a primary green energy source, driving substantial interest among the public, industry, and governments due to its future fuel potential. Notable investment is directed toward hydrogen research and material innovation for transmission, storage, fuel cells, and sensors. Ensuring safe and dependable hydrogen facilities is paramount, given the challenges in accident control. Addressing material compatibility issues within hydrogen systems remains a critical focus. Challenges, roadmaps, and scenarios steer long-term planning and technology outlooks. Strategic visions align actions and policies, encompassing societal and ecological dimensions. The confluence of hydrogen’s promise with material progress holds the prospect of reshaping our energy landscape sustainably. Forming collective future perspectives to foresee this emerging technology’s potential benefits is valuable. Our review article comprehensively explores the forthcoming challenges in hydrogen technology. We extensively examine the challenges and opportunities associated with hydrogen production, incorporating CO_2_ capture technology. Furthermore, the interaction of materials and composites with hydrogen, particularly in the context of hydrogen transmission, pipeline, and infrastructure, are discussed to understand the interplay between materials and hydrogen dynamics. Additionally, the exploration extends to the embrittlement phenomena during storage and transmission, coupled with a comprehensive examination of the advancements and hurdles intrinsic to hydrogen fuel cells. Finally, our exploration encompasses addressing hydrogen safety from an industrial perspective. By illuminating these dimensions, our article provides a panoramic view of the evolving hydrogen landscape.

## 1. Introduction

In the present day, energy and transport systems are significantly driven by petroleum fuels, which are not sustainable. The global demand for energy is expected to rise in the upcoming decades—by more than 50% until 2030, according to the International Energy Agency (IEA) [1]. In order to prevent anthropogenic interference with the climate system, the increasing greenhouse gas (GHG) emissions need to be reduced. From a diverse mix of energy sources, industrially, hydrogen can be produced in large quantities, which could be able to play a key role in the energy sector [2]. While hydrogen is used in fuel cells, it transforms the fuel’s chemical energy into electrical energy and produces water as a byproduct, which is considered zero emission. According to the Paris Agreement goal of keeping the temperature globally below 20 °C, world leaders, countries, and companies are moving forward to achieve net zero emission by 2050 [3]. Goldman Sachs’ research published reports about the rise of clean hydrogen and the clean hydrogen revolution [4]. Clean hydrogen has the potential to aid in the decarbonization of 45% of global anthropogenic emissions, according to the report [4]. Hydrogen can be utilized as an energy carrier in transportation, power generation, and chemical industries with zero carbon emission. The first Energy Earth shot, launched 7 June 2021—Hydrogen Shot—approaches to cut the expenditure of clean hydrogen by 80% to USD 1 per 1 kg in 10 years (“1 1 1”) [5]. The global market for hydrogen gas is expected to be around USD 154.74 billion by 2022 and keep increasing going forward [6,7].

In the following chart, hydrogen demand potentials in TWh are shown as 2030 and 2050 visions. The highest demand potential in the future will be the existing industry feedstock, where the US will increase its potential, whereas, in Europe, it will decrease. The biggest difference in the 2030 and 2050 potentials is in the transportation field, where the demand has grown exponentially in both the US and Europe [8,9].

**Figure 1:**
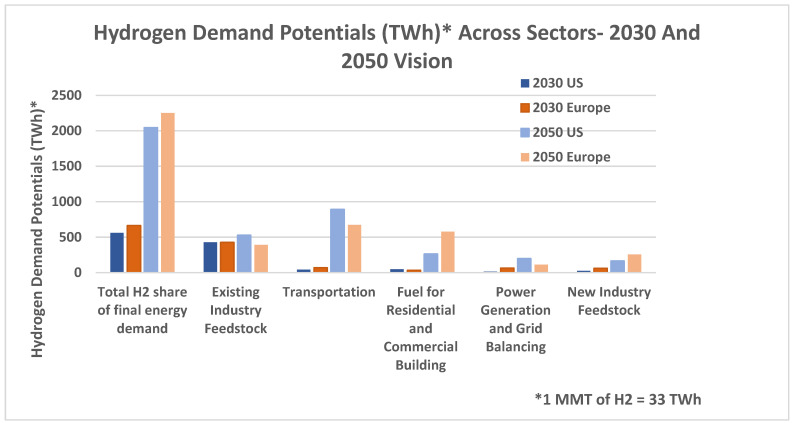
Hydrogen demand potentials (TWh) across sectors—2030 and 2050 visions [8,9].

In the announced pledges scenario, hydrogen is mostly used in sectors like industry and transport. In the second chart, the effect of rising renewable energy penetration becomes more pronounced as it is projected for net zero carbon emission. In terms of the environmental effect, the second scenario wins, but the announced pledges scenario paints a more realistic picture of where we might see the world in 30 years in terms of the hydrogen demand. Figure 1 illustrates the hydrogen demand potentials.

**Figure 2:**
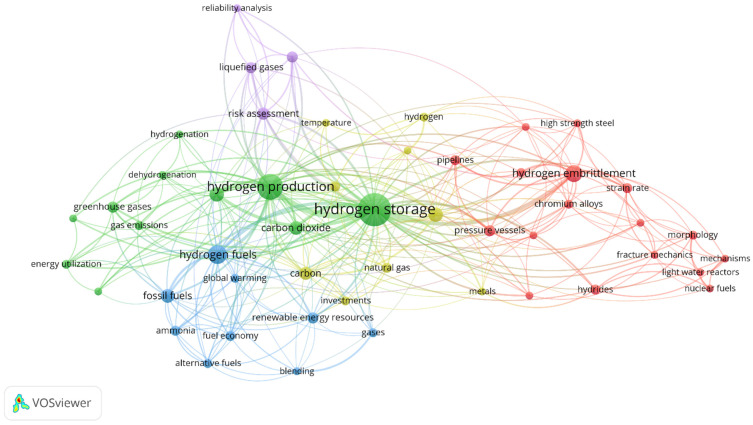
Cooccurrence of keyword networks for an overlay visualization map of hydrogen research developed by VOSviewer (https://www.vosviewer.com/, accessed on 16 September 2023).

The initial key step was to investigate the significant characters of a research field and identify the key terminologies. The density visualization method was used here to analyze the keywords using VOS viewer software. (version 1.6.19; access on 18 September 2023) The lowest number of recurrences of a keyword was set to 3. Of the total 300 keywords, only 50 were able to meet the threshold. Figure 2 indicates the diversity of the hydrogen research field and that it consists of interdisciplinary characters. From this figure, we can observe that the most recurrent phrases are hydrogen embrittlement, hydrogen storage, and hydrogen economy, illustrating the main current challenges. The “clusters” connected with phrases like hydrogen embrittlement, hydrogen production, transport, hydrogen storage, hydrogenation, combustion, and transportation represent the emission reduction strategy through hydrogen research. An interesting aspect of these emission reduction strategies is the focus on material properties and material interactions, as outlined by the recurrence and connections of phrases such as high-strength steel, alloys, morphology, pressure vessel, hydrogen, and hydrides, among others. These phrases are of particular importance, as they illustrate the current research aimed at improving the understanding of the sources for limitations in the current technologies as they relate to hydrogen transport, storage, and use.

Similarly, the term “hydrogen economy” is encircled by aspects such as hydrogen production, hydrogen storage, infrastructure, emission, and the hydrogen supply chain. This visibly directs the key areas that are incorporated to cover the range of hydrogen research. The timeframe indicates that recent research has focused on the challenges, hydrogen production, hydrogen transportation, power-to-gas, combustion, green hydrogen production, performance, safety, and life cycle assessment areas. For these reasons, this article presents an extensive review of the main material challenges for hydrogen compatibility and recommendations for future research directions around hydrogen. US Department of Energy report, and the relevant industry literature were referred to in order to perform this review research.

However, in order to establish hydrogen fuel on an industrial scale, there are still a number of challenges that need to be addressed. One of the main challenges for hydrogen gas is from a material perspective because of the nature of hydrogen gas. The behavior of hydrogen is not same as natural gas. For production, transportation, storage, fuel cells, combustion engines, and sensors, the design should be dedicated and specific for a hydrogen environment. Research groups have been working to identify or make new materials for hydrogen applications. The hydrogen embrittlement problem is the main key issue for hydrogen compatible materials from production, transportation, storage, fuel cells, and combustion engines to sensor applications. Industries including metals processing, chemical, mechanical, and refineries have difficulties with material degradation due to hydrogen exposure for many years. Hydrogen embrittlement is one of the oldest and most frequently observed degradation phenomena in production, storage, transportation, and distribution facilities. Several theories have been explained based on the sources of hydrogen and types of embrittlement. It can be classified into three types: (I) hydrogen reaction embrittlement, (II) internal reversible hydrogen embrittlement, and (III) hydrogen environment embrittlement [10].

## 2. Hydrogen Production

**Figure 3:**
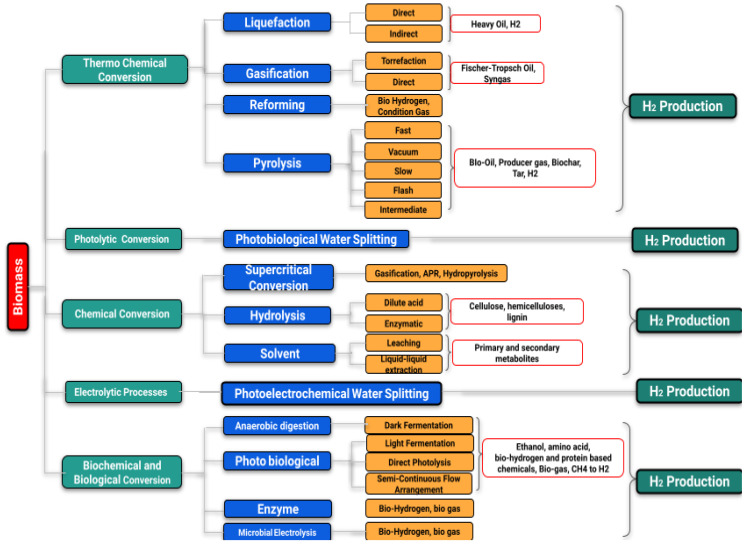
Illustration of different technologies of hydrogen production, adopted from [11].

Hydrogen can be produced from numerous sources (e.g., fossil fuels, biomass, and water) by using several technologies. However, the production of H_2_ has dominated using fossil fuels until today. Day by day, the water electrolysis method has gained more interest with the decreasing renewable power cost for green hydrogen production. Nearly 76% of total hydrogen gas (of the global demand) is produced from natural gas sources. For the rest, 23% and 1% are from coal and electrolysis sources, respectively. The amount of CO_2_ emission during hydrogen production has been a concerning issue for a while, and efforts are continuing to control CO_2_ emission. The implementation of carbon capture, utilization, and storage (CCUS) projects are helpful in this regard. Numerous types of routes for hydrogen production are outlined in Figure 3.

Sources and methods of gaining different types of hydrogen, along with the emission types, are listed in Table 1.

Green hydrogen is achieved through the process of electrolysis powered by renewable energies such as wind or solar. Electrolysis involves using an electrical current to break down the water molecule into oxygen and hydrogen by electrodes. Hydrogen stored in specific tanks is channeled into a fuel cell. There, it binds again with oxygen from the air, and electricity is obtained. Thus, the only byproduct of the process is water, resulting in a clean, sustainable system in which zero CO_2_ is emitted to produce energy. In the study by Marcelo and Dell’Era [15], they identified two primary categories of electrolysis processes: (i) polymer electrolyte membrane (PEM) electrolyzers and (ii) alkaline electrolyzers. The operational efficiencies of these electrolyzers can vary from 52% to 85%, as reported by Binder et al. in 2018 [16]. The conversion of electricity into chemical energy through electrolysis represents a promising technological advancement. This explains the global proliferation of power-to-gas facilities. Proton exchange membrane (PEM) electrolyzers and alkaline electrolyzers, as well as other electrolyzer types such as solid oxide electrolysis cells (SOECs) and molten carbonate electrolysis cells (MCECs), have gained widespread recognition for their electrochemical applications.

To establish the financial feasibility of a large-scale production unit, an energy analysis is essential. This analysis can be conducted using the standard methods [11,17]. It involves calculating the input energy (Ei), output energy (Eo), and net energy (En) using the following equations:

The input energy (Ei) can be determined using Equation (1):

Ei = P ∗ T ∗ V ∗ S(1)

where Ei represents the input energy (kWh), P is the power used for the process (kW/kg), T is the time for disintegration (hours), V is the reactor volume (m^3^), and S is the substrate (kg/m^3^). This energy is required for the disintegration process.

The output energy (Eo), in terms of energy gained as hydrogen, can be calculated using Equation (2):

Eo = B ∗ L ∗ H ∗ V ∗ F(2)

where Eo denotes the output energy (kWh), B is the biodegradability of algal biomass (gCOD/gCOD, where COD is the chemical oxygen demand), L is the COD load (gCOD/m^3^), H is the hydrogen yield (m^3^/gCOD), V is the reactor volume (m^3^), and F is the biohydrogen conversion factor (1 m^3^ equals 3.5 kWh).

The net energy (En) estimation is determined by the difference between the output energy (Eo) and input energy (Ei) using Equation (3):

En = Eo − Ei(3)

where En represents the net energy (kWh), Eo is the output energy (kWh), and Ei is the input energy (kWh) [18].

This energy analysis is crucial for assessing the financial viability of large-scale production units.

### 2.1. Hydrogen Production from Natural Gas: Challenges and Opportunities

The most universal method to produce hydrogen from natural gas resources is known as steam methane reforming (SMR). At present, three methods are in use for hydrogen production from natural gas resources. These are SMR, the partial oxidation method (POM), and autothermal reforming (ATR). For large-scale production, SMR is the most popular form of H_2_ production [19,20]. CO_2_ produced by the SMR method is currently released into the atmosphere. However, it can be utilized as a byproduct in food processing and packaging. SMR uses water as an oxidant and a source of hydrogen, while oxygen in the air is used as an oxidant in POM. A combination of both SRM and POM is known as the ATR method. SMR is the most developed industrial process with no oxygen requirement. However, the emission of CO_2_ is considerably high. With a view toward decarbonizing these methane-based processes, the CO_2_ produced must be captured and stored. The use of carbon capture, utilization, and storage (CCUS) projects are helpful in this regard. It is a process that captures carbon dioxide emissions from different sources so that it will not enter the atmosphere. CCUS projects involve pumping the CO_2_ into geological reservoirs, such as depleted oil and gas fields [10,11,12]. Being a sustainable method with a low current cost, SMR has become popular in H_2_ production. However, difficulties involved with the automated control system and feedstock management system need to be addressed to make SMR a more efficient way of producing H_2_.

### 2.2. Electrolysis: Challenges and Opportunities

Electrolysis is an electrochemical process where less than 0.1% of the dedicated hydrogen production becomes global [8,21,22]. Three main electrolysis technologies include alkaline electrolysis, proton exchange membrane (PEM) electrolysis, and solid oxide electrolysis cells (SOECs). Fertilizer and chlorine industries have used alkaline electrolysis to produce hydrogen since 1920, and it is as an established and commercial technology. General Electric introduced proton exchange membrane (PEM) electrolysis in 1960 to overcome the few operational drawbacks of alkaline electrolysis. The least-developed electrolysis technology is solid oxide electrolysis cells (SOECs), which are yet to be commercialized. Integrating heat into hydrogen production became interesting because of the heat sources from industrial processes, geothermal or solar heat, and nuclear power plants. Nuclear power plants generate heat at 300 °C, which can also be used to provide electricity and steam for solid oxide electrolysis cells (SOECs) [8,23].

Electrolysis requires both water and electricity. Approximately 9 L of water are used to produce 1 kgH_2_, while 8 kg of oxygen are produced as a byproduct. As a matter of fact, freshwater access can be an issue in water-stressed areas, as water consumption is roughly double that of the SMR process. Seawater could be a potential source for electrolysis in coastal areas. But seawater may not be used directly, as it leads to corrosive damage and the production of chlorine. Moreover, PEM electrolysis needs expensive electrode catalysts such as platinum, iridium, and membrane materials. Again, the lifetime of the electrodes are also shorter than those of alkaline electrolyzes [8,21,22]. A fuel cell is relatively dry compared to the electrolysis process. Water is soaked by polymer electrodes and therefore swells. During extreme swelling and operating conditions at 30 bar (430 psi) or higher, polymer strands are farther apart and become weaker mechanically without the use of thick membranes ranging from 175 to 250 microns.

Having mentioned these limitations, there are new opportunities that appear every day to mitigate the gaps. Electrolysis cell efficiency may be improved by using thinner membrane materials (50–60 microns). It also assists to increase the mechanical strength [24]. With a view toward optimizing ionic conductivity and decreasing water uptake, researchers are investigating different polymer compositions. The replacement of the fluorine-based backbone of Nafion with hydrocarbons has been taken under consideration. Also, the catalysts used in electrolysis—platinum on the hydrogen side and iridium on the oxygen side—need to be improved [24]. Research on selecting materials that would be compatible with the temperature levels of nuclear energy heat sources needs to be performed. Small modular reactors will also be considered to contribute to SOEC electrolysis in the coming days. Also, advanced nuclear reactors will be an attractive option as well in the long term [8,23].

### 2.3. Hydrogen Production from Coal: Challenges and Opportunities

The chemical and fertilizer industries produce hydrogen from coal using the gasification process during the production of ammonia as a well-established technology [25,26]. However, the CO_2_ emission increase is very likely from coal-based hydrogen production. Also, the use of CCUS produces hydrogen with a relatively low hydrogen-to-carbon ratio and can contain high levels of impurities in the feedstock (sulfur, nitrogen, and minerals) [25,26]. Heat released from the gasifier unit needs more efficient uses, including power generation and heating purpose. However, it is also challenging to cool hot gas above 1400 °C using heat exchangers due to material restrictions. The other challenge is the lack of established standard codes. The carbon content carried in syngas should be reduced to avoid greenhouse emission.

Coal contains sulfur, which results in the production of sulfur oxide during the gasification process. Sulfur oxide is responsible for environmental pollution and acid rain. Therefore, a sulfur reduction system must be incorporated into the gasification process of coal for a sustainable environment. The devolatilization of coal can be carried out by using different types of fuel. Also, the devolatilization temperatures can be integrated into the segregation of coal. Waste heat and hydrogen may be further used in power generation systems, storage, and cooling purposes by the application of the plasma co-gasification process.

### 2.4. Hydrogen Production from Biomass: Challenges and Opportunities

There have been numerous ways to produce hydrogen from biomass. One of the routes is called the biochemical route, where biogas is generated by the interaction of a microorganism with an organic material. The process is also known as aerobic digestion, where a combination of acids, alcohols, and gases takes place. Microorganisms, such as bacteria, break down organic matter to produce hydrogen. The organic matter can be refined sugars, raw biomass sources such as corn stover, and even wastewater. Because no light is required, these methods are sometimes called “dark fermentation” methods. In direct hydrogen fermentation, the microbes produce the hydrogen themselves. These microbes can break down complex molecules through many different pathways, and the byproducts of some of the pathways can be combined by enzymes to produce hydrogen.

Although several biomass gasification plants exist in the world, the technology is not yet fully established. Catalyst poisoning resulting from the formation of tar has not been completely addressed. Irrespective of the production process, the produced gas needs to be further processed to extract hydrogen. The unavailability of cheap biomass also restricts biomass-based hydrogen production on a large scale [11]. Converting hydrogen to hydrogen-based fuels and feedstocks is easier to store, transport, and use. Some examples are ammonia, synthetic hydrocarbons, and synthetic methanol [27,28].

### 2.5. Methane Splitting: Challenges and Opportunities

Around 1990, the methane splitting process was introduced, which is based on alternating the current three-phase plasma. It uses methane as feed and electricity as the energy source to produce hydrogen and solid carbon without the emission of CO_2_ from natural gas [27,28]. The methane splitting process consumes electricity three to five times less than electrolysis for the same amount of hydrogen production. However, the process comes with limitations, because it requires high-temperature plasma and a significant loss of temperature, which reduces the overall efficiency [27,28].

Monolith Materials operates a pilot methane splitting plant in California and is building an industrial plant in Nebraska with a lower total efficiency than using natural gas directly in the power plant. This process can help reduce the emissions from gas combustion, and even if it requires more natural gas than electrolysis, there could be additional revenue streams from the sale of carbon black for use in rubber, tires, printers, and plastics [29,30].

## 3. CO_2_ Capturing Technology in Hydrogen Production

Technologically advanced gray hydrogen production methods are facing questions about CO_2_ emission. However, CO_2_ emission reduction could be possible up to 90% by using carbon capture, utilization, and storage (CCUS) in the system. Lots of challenges need to be overcome for optimizing the demand with an environmentally friendly efficient hydrogen production pathway globally. A significant amount of CO_2_ emissions have been observed while producing H_2_ from different sources. Although several CO_2_ capturing technologies have been explored for the SMR and ATR methods with a view toward increasing the efficiency with low costs, more research is still required for implementing CO_2_ capture technology during H_2_ production from natural gases and coal sources. In a study, the regional H_2_ production cost from natural gas was found to be less than USD 1/kg of H_2_, including the natural gas cost, OPEX, and CAPEX [12,13,14].

Being an energy carrier, hydrogen stores and delivers energy in some usable form. As discussed, we can produce hydrogen from diverse sources, such as natural gas, biomass, renewable energy, hydroelectric power, or nuclear energy sources. The diverse supply sources make hydrogen a promising energy carrier. Also, it has the flexibility to be produced in large, medium, or small distributed units located in proximity to the consumer, such as refueling stations or stationary power sites. Therefore, extensive research is being carried out to produce commercially viable hydrogen from different sources. At the same time, technologies should be developed in capturing CO_2_ emissions to increase the efficiency with low costs during H_2_ production from natural gases and coal sources. A visible development in technologies can be observed for both the steam methane reforming and partial oxidation methods, and both have been used commercially for producing hydrogen. Accordingly, it is also required to develop the delivery methods and improve the infrastructure to ensure minimum or no greenhouse gas emissions from coal-based hydrogen production. Furthermore, improving biomass growth, harvesting, and handling to reduce the cost of biomass resources used in hydrogen production is also necessary.

Carbon sequestration technology to mitigate climate change during hydrogen production has been long discussed. During post-combustion, CO_2_ is captured by chemical absorption using monoethanolamine (MEA). High-purity CO_2_ is produced from the exhaust of coal- or gas-fired boilers using this technology. The regenerated absorbent is recycled to the absorber, and CO_2_ is dried and compressed for transport conditions (typically between 100 and 150 bar).

Adsorption, low temperature distillation, and membranes can also be utilized to capture CO_2_ from flue gas. The physical adsorption of CO_2_ at a solid surface (zeolite) is highly energy-consuming at conventional pressures and temperatures. Using commercially available polymeric membranes results in relatively large energy requirements and CO_2_ avoidance costs in comparison to chemical absorption, due to the low driving force as a consequence of the low CO_2_ partial pressure in flue gas [31,32].

A summary of the challenges and opportunities of selective hydrogen production methods, along with their current research and development status, is shown in Table 2.

## 4. Materials and Composites for Hydrogen Transmission, Pipeline, and Infrastructure

From a material point of view, hydrogen gas faces challenges due to its characteristics and reactivity with the overall infrastructure, including transmission, pipeline, storage, and production facilities. Embrittlement is the classic major problem for hydrogen infrastructures encountered by researchers since long ago. Also, an energy-dense and reactive gas such as hydrogen leads to polymeric material challenges in the infrastructure presently for its safe operation.

The purity of hydrogen is a key factor for hydrogen rupture pressure. Oxygen or traces of water vapor can partially inhibit the hydrogen embrittlement effect [35]. Usually, the purer the hydrogen, the more embrittlement is observed. The reason is likely that impurities like SO_2_ in hydrogen have an inhibiting impact on embrittlement. Again, other impurities such as CH_4_ and N_2_ do not seem to have any appreciable effect. On the contrary, there are some impurities like CO_2_ and H_2_S that have an adverse effect on hydrogen-induced embrittlement [35]. The microstructure of steel and other materials resulting from heat treatment and chemical composition also plays a significant role in HE. It has been observed that martensitic structures have the worst behavior, ferritic structures an intermediate behavior, and stable austenitic steels exhibit the best behavior [35]. The welding of steel is also a major parameter that can greatly affect HE behavior.

The hardness of the welded area and heat-affected zone should be limited to carbon steels. The formation of ferrite should be limited in austenitic stainless steel [35,36]. Ni, Ti, and high Ni alloys and Ti alloys are very sensitive to HE. Again, HE behavior may remain even at rather high temperatures of steel materials. The impact of high-pressure and high pure hydrogen is also evident on metals. Depending on the material, the HE increases upon increasing the pressure. Austenitic steel is considered the safer option for HE [31]. Hydrogen atoms enter into the iron sample once it is subjected to stress or tension above the elastic limit. From the analysis of the crack formation, it has been observed that, under normal operating conditions, the change in pressure does not cause failure; while the pipe is affected by HE, it will not be able to take the pressure for a long time and crack [37].

It is very sensitive to calculate the fatigue life while there are growing crack, defect, and infrequent pressure cycles. The predicted lifetime of a pipeline for a hydrogen–natural gas blend and for 100% hydrogen is about the same: many decades [38]. Experimental studies have predicted the lifespan of steel under high-pressure hydrogen effects. The estimated lifespan of X70 API 5L grade steel is more than 50 years [38]; the API steels (X52, X60, X65, X70, and API 5L Grade B) and X42 steel lifespans are also determined to be more than 50 years with different load rations R [39]; and the X80 steel lifespan is estimated at 37 years with a 50% hydrogen gas mix with natural gas [40]. These lifespan estimations are based on assumptions for two pressure cycles per day.

The permeability of hydrogen increases when increasing the hydrogen pressure, but the ratio gets slower with the further increasing of pressure. All the permeability, diffusion, and solubility coefficients are correlated with a specific volume in high-pressure environments. The gas diffusion decreases upon the compressive effect of the free volume by the application of hydrogen. The permeation coefficient also decreases with the pressure increase from the measurement of the crystallinity; it is evident that gas penetration takes place in an amorphous region under a high-pressure hydrogen environment [38]. The degree of destruction is greatly influenced by the quantity of the gas penetration. However, the type of material is also a significant factor to determine the damage.

### 4.1. Polymers Used in Hydrogen Application

The applications of polymer materials are vast in the hydrogen industry. They are used for seals in connections, compression equipment, tubing, dispensing, and valves, as well as the lining of the storage cylinders. Four classes of polymer materials are used in hydrogen applications based on their microstructure. These are semicrystalline thermoplastics, amorphous thermoplastics, elastomers, and epoxies [41,42,43].

Leaks at critical interfaces, such as onboard tank systems, the dispenser–vehicle interface, and supply systems, are very common. O-rings (elastomeric) are widely used in the hydrogen industry in both static and dynamic designs to prevent leaks in systems [44]. Transient conditions must be considered during designing the specification of a static seal. Temporary leaks can take place in elastomeric materials during the motion of a shaft in valves or regulators. In the case of dynamic seal designs, the issues can result in a temporary leak during movement between the sealing surfaces.

The tribology of polymers plays an important role in dynamic sealing applications. A longer lifetime for polymers in a high-pressure hydrogen environment depends on the low coefficient of friction, which means less wear and tear. Depending on the permeation characteristics of the polymers, the friction behavior can differ. Fillers in polymers can play a large role in increasing or decreasing their susceptibility to hydrogen [45]. The effects of plasticization, fracture, and fatigue should be kept in mind while selecting polymers for a high-pressure hydrogen environment. Additionally, the rapid gas decompression of elastomers with the combined effects of temperature and pressure cycling metrics for assessing the damage and mitigation through the development of damage-resistant materials needs to be studied as well [45]. Thermoplastic and elastomeric polymers have been studied under high-pressure hydrogen (70–100 MPa) in static, isothermal, and isobaric conditions to characterize the physical properties and perform a mechanical analysis. During fuel consumption and refueling operations of fuel cell vehicles, polymers are exposed to a large pressure gradient. In dynamic environment such as in the high-pressure cycling of hydrogen (35 MPa to 100 MPa to 35 MPa), the performance of these polymers is highly influenced [41,45].

Picking up the right sealing material for the hydrogen flow is one of the most crucial challenges to prevent gas permeation and absorption. While characterizing polymeric materials, several things need to be considered. The measurements of crystallinity, degree of polymerization, crosslink density, outgassing and desorption of chemical species, permeation/absorption of hydrogen into polymers, gas exposure time, temperature, pressure, pressurization/depressurization rates, durability, correlation of bench-scale (coupon) versus full-scale material characteristics, etc. are important [44]. The effects of hydrogen, temperature, and pressure on “creep” and “strength” need to be investigated. Also, plasticization effects such as a change in ductility under a hydrogen environment have yet to be explored. Understanding the supercritical properties of hydrogen as a solvent on a polymer is important to customize the appropriate polymer selection.

#### Challenges and Opportunities with Polymers

Polymers, whether used in seals, compressors, tubing, or pressure vessels, can fail in a variety of ways. Although hydrogen embrittlement mechanisms in metals appear to be absent in polymers, exposure to high-pressure hydrogen does affect the mechanical performance of polymers. Being one of the smallest molecules, hydrogen can diffuse into and through polymers much more easily than any other gases. While operating in hydrogen, some polymers can maintain their properties. However, the behavior of different polymers under a hydrogen environment is yet to be explored.

When hydrogen absorbs into and diffuses through polymer components at high pressure, it forms small bubbles within the material, causing damage. If the depressurization is substantial and rapid enough, these bubbles can exit the polymer violently, causing substantial damage and failure of the component. This rapid gas decompression can occur even at a timescale of hours, meaning this effect is not limited to sudden and drastic changes in pressure.

While the hydrogen effect on a few selective polymers, including EVA, PVC, EPDM, etc. have been studied, a large portion—thermoplastics, in particular—have not been thoroughly investigated for this failure mechanism. Also, the high pressure used to store hydrogen can have some effects on the plasticization of the polymers, which needs to be studied as well. Extensive research on the fracture and fatigue failure modes, rapid gas decomposition, friction and wear, test methods, plasticization, transport properties, and contaminants is necessary [41,42].

### 4.2. Composites Used in Hydrogen Systems

Composites used as hydrogen storage materials are different. Kevlar and glass fibers have been shown to be more damage-tolerant than carbon fibers. Composite cylinder designs have recently improved to become more damage-resistant by adding additives to damage-sensitive areas and adding sacrificial layers to the outside of the entire tank [46]. Carbon fiber manufacture needs economic and advanced material characterization methods to monitor the key performance. The key performance properties include the defect structure, resistivity change during carbonization, density change during oxidation, etc. Screen alternative fibers are also considered as potential candidates in the application of filament winding [41,42].

#### Challenges and Opportunities with Composites

Stress rupture is an important but not fully understood problem with composite tanks. It occurs when multiple fibers randomly fail next to each other. Normally, when a single fiber fails, the surrounding fibers can hold the load that was previously carried by the failed fiber. However, when multiple fibers fail very close to each other, the surrounding fibers are suddenly put under a much greater load, leading them to fail and so on, until the entire vessel/layers rupture. This failure has little or no advanced warning and can lead to catastrophic results. One way these fibers may fail is due to corrosion, such as the oxidation of carbon fibers. This mechanism has been studied under various atmospheres (including air) and at various temperatures, and it was found that high-temperature samples in air had the highest rates of failure. One problem with the study of stress ruptures is the inherently stochastic mechanism they follow. Fibers can fail more or less randomly, and so, it is inherently random if a sufficient number of fibers happen to fail in a cluster close to each other. This means that experimental studies of this phenomenon need high numbers of samples, typically with high lifetimes, in order to produce failures [47].

Understanding the material properties of composites and polymers is challenging when there are complex operating conditions with multiple variables. For example, at a wide range of temperatures as low as (−40 °C) to as high as (85 °C) with high pressure (700 bar), it is difficult to interpret the composite behavior on hydrogen. Also, there have been localized thermal extrusions during refueling or defueling operations of hydrogen-fueled vehicles. The impact of localized thermal extrusion is yet to be explored. Specifications such as SAE J2601, SAE J2579/GTR, CSA HGV 4.3, CSA HGV 2, and ISO 15869 could be incorporated into the fueling standards. A correlation between the material behavior and degradation at thermal soak and in-service temperature excursions needs to be established for certification. A temperature gradient exists between the gaseous hydrogen and sealing material during the fueling or defueling operation. The temperature difference must be considered to correlate with the test results from the thermal soak to in-service condition [41,42].

In order to determine the durability of a composite material, cycling loading experiments are performed rather than material tests on the constituent polymers. Cycles of around 90 MPa hydrogen pressure are usually maintained for the durability test. The crack damage and extrusion fracture of the composites are observed at various temperature, pressure, and cycling conditions. The crack damage was noticeably worsened by increases in the pressure (from 35 to 70 MPa) and temperature (from 60 °C to 100 °C) at the EPDM O-ring [48,49]. The key advantage of composite pressure vessels is the lesser weight. However, the aging of a carbon-fiber-reinforced plastic (CFRP) composite pressure vessel underlies the complex interaction with its metallic liner, which is yet to be understood. The appropriate testing methods to detect the influence of the pressure cycle and creep behavior on the material are yet to be established. A hydraulic internal pressure test usually cannot detect cracks unless fully developed through the metallic liner, whereas ET can be a promising alternate test. High-frequency ET can also be used to investigate the aging behavior of composites. Usually, the eddy current is introduced to detect cracks at high-frequency regions of fibers. Also, signals coming from the resin are more detectable in high-frequency applications. Composite overwrapped pressure vessel (COPV) failures, Kevlar, and glass fibers have been shown to be more damage-tolerant than carbon fibers [50].

### 4.3. Hydrogen Compatible Metal: Research and Challenges

The probability of a hydrogen attack mostly depends on the metallurgical structure of the steel. The chemical composition, distribution, and morphology of the phases; grain structure (size, shape, and texture); segregation; and distribution of intentional alloying elements and precipitates, as well as impurities, are the factors that change the metallurgical structure. However, the effect of the fundamental metallurgical reason behind using lower-strength steels over higher ones in existing natural gas pipelines, which have already been proven historically, is still doubtful [51,52]. Modern PE pipes enable the transport of hydrogen. Polyethylene pipe, PE80 and PE100, has several significant advantages over traditional materials such as steel or ductile iron. The permeation of contaminants in PE 80 and 100 materials explore the relationships between crystallinity and permeability, diffusion, and partitioning. The dominating factors to assess the hydrogen compatibility of metal are the structural changes in the polymer, consumption of antioxidants, change in the tensile properties, change in the slow crack growth properties of the material, surface oxidation, and metering calibration.

The test apparatus developed by NIST can measure 10 specimens simultaneously with specific environmental and loading conditions until all of them have been tested to be used in the industry. Over 150 fatigue tests were completed by them on base metals, welds, and the heat-affected zones of candidate steels. A modification was done to the ASME B31.12 by NIST to permit the use of X70 steel rather than X52 steel, which would result in a savings of over USD 1 million per mile of pipeline [53,54]. The standard guidelines like ASME B31.12 and the AIGA/EIGA permit X80/L555-graded materials to be used in hydrogen service. But ASME B31.12 explicitly states that only grades up to X52/L360 are proven for hydrogen gas services. The destructive testing of material samples at a minimum frequency of one sample per mile is recommended by the existing codes. The RoMat family was introduced by ROSEN for inline inspection services that include pipe grade sensor (PGS) technology and DMG hard spot technology. The aim is to support operators through the processes of material verification [52,55,56].

The US natural gas pipeline system states the feasibility of using relatively less concentrated hydrogen (5–15% by volume) with some minor modifications to the existing pipeline systems. To blend 10% hydrogen with a pressure drop ranging from 300 psi to 30 psi, the extraction cost varies from USD 0.3 to USD 1.3 per unit kg of hydrogen. A higher concentration of hydrogen demands structural modifications of the carrier. At the same time, the impact on end use systems, safety, material durability and integrity management, and the leakage rate need to be considered [57]. A comparative review of the hydrogen storage capacities of different materials is shown in Table 3.

## 5. Hydrogen Embrittlement

Hydrogen embrittlement is recognized as the most common event in the production, transportation, storage, and utilization of hydrogen. As a matter of fact, research on hydrogen embrittlement behavior under a high-pressure hydrogen environment has been going on for a long time. It can degrade the material properties significantly while making it weak. And this is done by the ingression of a hydrogen atom in or below the metal surface.

Hydrogen embrittlement is very frequent in petrochemical industries. When absorbed by metal, hydrogen can react to form a new phase near metal surfaces or diffuse substantial distances within the metal to produce a hydride (MHx) phase. During steel processing and welding, molecular hydrogen (H_2_) can be formed within the metal if atomic hydrogen reacts with itself, creating flaking or fisheyes. In low-alloy steels, hydrogen reacts with carbon to produce methane (CH_4_) bubbles. A safe operating condition for steels in hydrogen environments can be represented by Nelson diagrams [10].

If hydrogen does not produce any chemical reaction after being absorbed and diffused within the metal lattice from hydrogen sources, then slow strain rate embrittlement and delayed failure occur. For steels, embrittlement is usually most severe at room temperature. The extent of hydrogen diffusion within the lattice controls this type of embrittlement. Extensive focus has been given to the problems related to embrittlement that occur during electroplating, particularly of cadmium on high-strength steel components. Internal reversible hydrogen embrittlement has also been observed in a wide variety of other materials, including nickel-based alloys and stainless steels [10].

Hydrogen embrittlement has been considered a serious concern since 1960. The embrittlement magnitude has been analyzed over a wide range of operating variables, including gas pressure and temperature, through different mechanical tests. At room temperature, embrittlement was found to be the maximum. The purity of the gas and the test strain rate are also significant parameters to determine the extent of the embrittlement. Quantitative analyses indicate substantial increases in the hydrogen content of embrittled alloys. The position of the crack initiation, whether from the surface or from the inside, is yet to get explored [10].

### 5.1. HE during Hydrogen Production

Hydrogen is considered the heat transfer medium during hydrogen production, coal gasification, or advanced energy conversion processes. From a pure or hydrogen-rich environment, hydrogen is transported to samples like steel and low-alloy steels. Temperature, pressure, and applied stress are the variables to be investigated for the interaction of hydrogen with the steels. The other variables that can be considered are modified alloy composition, modified microstructure of the steel, and chemical modification of the environment. The mechanical properties are controlled by the mechanism of hydrogen exposure to the steel at ambient and elevated temperatures. This interaction is yet to be explored. This would be of significant benefit not only for coal gasification processes but also for hydrogen production processes [10].

### 5.2. HE during Transmission/Storage

The transportation medium of hydrogen has been considered in a gaseous, liquid, or even as a solid hydride phase. The storage and transportation facilities of hydrogen are designed in a way that are most likely free from environmental degradation. However, the design comes with limitations, as it needs expensive alloy systems such as chromium and nickel [10,65].

### 5.3. HE during the Transmission/Storage of Liquid Hydrogen and Hydride

Liquid hydrogen storage and transport still remain challenging. The reaction processes, including the dissociation of molecular hydrogen, are very slow at cryogenic temperatures. Therefore, the transport of hydrogen from the environment into the metal becomes very slow and difficult. For example, the failure to select appropriate materials, insufficient galvanic corrosion protection, or improper protective coating results in the corrosion of stainless steel and associated tools. Furthermore, if there is the application of thermal cycling where hydrogen penetrates the metal at a high temperature, then the problems are more severe. The underlying reason is the formation of a second phase, either gaseous or solid hydride, which leads to embrittlement. Hydrogen-induced embrittlement causes a loss of integrity of the storage and transport system. Stable austenitic stainless steels or aluminum alloys are widely being used for liquid hydrogen transport or storage systems [65,66]. Gaseous and liquid hydrogen storage systems differ significantly in terms of hydride formation. The pressure, temperature, and thermal cycling are considered in the operating design. In addition, the role of atomic hydrogen in the hydrides on the embrittlement of containment materials is unresolved [65].

### 5.4. HE during the Transmission of Gaseous Hydrogen

Hydrogen embrittlement is a big concern to pressure vessels and pipelines due to insufficient galvanic corrosion protection, poor welding quality, and hydrogen embrittlement-susceptible steel materials. Low-strength steels, poor welding, and low hydrogen pressures such as 0.3–7 MN/(50–1000 psi) are also obvious reasons for promoting hydrogen-induced embrittlement. Embrittlement is also likely due to high-strength steels under high-pressure hydrogen [10]. However, the evaluation of welded pipeline steel and candidate compressor alloys under simulated service conditions, improved welding, and nondestructive inspection technologies determine the feasibility of composite pipelines, and the compatibility of materials in corrosive environments needs to be studied extensively. Table 4 shows the commonly used materials and their embrittlement assessment. 

## 6. Hydrogen Fuel Cell and Combustion Engine for Hydrogen 

### 6.1. Challenges of Utilizing Hydrogen in Fuel Cells

Hydrogen has been considered a prospective candidate of combustible fuel because of its wide range of flammability, low ignition energy, small quenching distance, high autoignition temperature, high flame speed at stoichiometric ratios, high diffusivity, and very low density. But it comes with a number of challenges like backfire, auto, and pre-ignition. The relatively smaller molecular structure of hydrogen makes it difficult to store.

Blending hydrogen with other gases is recommended to make hydrogen tolerable for storage and transport, but the absence of a light, safe, and low-cost storage technology is still a bottleneck for hydrogen storage [68].

Fuel cells play a vital role in hydrogen. Starting from the production of hydrogen to direct uses in the automobile sector, fuel cells are involved. Most technologically mature fuel cells in the present days are a proton exchange membrane (PEM). Other than that, SOFC has shown significant potential in terms of efficiency for hydrogen applications, but it is not mature enough yet for commercial purposes. Despite having advantages in fuel cell uses, this technology also has limitations that need to be addressed. Hydrogen can also be used directly in combustion engines for various purposes like power generation, the aviation industry, and the automobile sector. Due to the chemical characteristics of hydrogen and rection behavior, combustion engines need modifications for a longer and safe service life. The literature supports hydrogen internal combustion engines (HICEs)/combustion engines for power generation or vehicle application development from giant companies.

### 6.2. Hydrogen Fuel-Based Vehicles: Pros and Cons

Hydrogen fuel-based vehicles have been developed by Toyota, which are powered by 13 engines of 1.6 L turbocharges each. The storage tank is 10 K psi. Homogeneous charge compression ignition (HCCI) is used for hydrogen combustion. The very lean hydrogen combustion of HCCI results in an emission of NOx only 1 ppm and 45% thermal efficiency achieved. Nitrogen in the air reacts with oxygen and hydrogen and forms NOx. Also, a small amount of CO_2_ is also generated from engine oil combustion [69]. The challenges with HCCI are that it is very difficult to control. The cold start compression ratio is about 42:1, and a typical gasoline engine compression ratio is 8:1 to 13:1, 25% less load capability than gas engine. That means one-fourth of the power [69,70,71,72]. HCCI has about 38% more efficiency than SI or PFI engines in terms of knocking, pre-ignition, backfiring, and quenching. HCCI still needs to improve compared to a typical gas engine in term of the gas load. Toyota is using turbo chargers to overcome this gas load problem. 

The Toyota Mirai has a 50% efficiency using fuel cells at the wheels, where hydrogen combustion is 25% efficient at the wheels. Therefore, fuel cells and combustion engines may be combined to exceed the optimization. But, due to energy density, it is not easy to convert existing engines to hydrogen fuel engines. The energy density for H_2_ is 1.3 kwh/L @ 700 bar and 9 kwh/L for an E10 engine. The storage size is seven times more for the same amount of energy and needs twice the tank size to overcome the lag between FC and ICE efficiency [70,72].

### 6.3. Incorporation of Hydrogen-Based Power in Industries

Hydrogen can be used as an alternate source of power. Manufacturing, chemical, and petrochemical industries that generate gas also produce hydrogen as byproducts. The excess gas can be utilized to operate a gas turbine that has already been adopted by GE for a decade. Ge developed the DLN 2.6e combustion system, which has the capability to burn fuel blends up to 50% hydrogen, and this combustion system was applied to the 7HA and 9HA gas engine series. Hydrogen is three times lighter than natural gas, which provides a higher flow rate. But the heating value of hydrogen is much lower: ~274 BTU/scf (10.7 MJ/Nm^3^), which requires configuring the fuel accessory system to accommodate the increased flow. A process stream containing a significant level of hydrogen may require the use of a diffusion flame combustor [73,74]. GE was able to lower the NOx emission by adopting a technology where fuel is charged to a GT chamber followed by a ring manifold before being delivered to each combustion chamber through “pig tails”. The homogeneous distribution of fuel is ensured by using four 1/2 in. distributing tubes attached on the outside of the “unibody” and then injecting it into the hot gas path. Therefore, fuel gas is introduced upstream of the turbine first stage nozzle, which is downstream of the flame zone. Thus, the fuel gas auto-ignites, increasing the energy available for the power turbine but with no increase in NOx emissions [75]. 

### 6.4. Hydrogen Fuel Usage in the Power Industry: Challenge and Research

While hydrogen has been used extensively in the power industry as fuel, it still comes with several challenges. A higher flame temperature and lower heating value of hydrogen leads to increased NOx production and an additional accessory system to accommodate the increased volumetric flow, respectively. The hydrogen flame speed is much faster than methane. Not only pure hydrogen but also the blend of 50% hydrogen and natural gas also have an increasing flame speed, which is, at minimum, double that of pure methane. The higher speed of the flame results in a higher risk of flame propagation into the pre-mixer. While the flame can stabilize and anchor inside the pre-mixer, it is known as flame holding. Both situations can lead to combustion hardware distress and even fuel nozzle damage [75].

The capacity is bounded to 5%, as the flame speed of hydrogen is high enough that it results flash back or reignition in the primary zone. While there is no loss of water, 1 unit of hydrogen consumes 9 units of water during electrolysis. The volume of hydrogen can differ from 35% to 85% based on the size of the turbine from 100 Mwe to 18 Mwe [75]. Therefore, theoretically, it is possible to compute the volume of water required to generate power from a hydrogen concept. Supplying that pool of water is a huge challenge. When a gas turbine is operated with the blend of hydrogen and natural gas rather than pure hydrogen, the water consumption also is reduced for producing less hydrogen. When a 9F.04 gas turbine is operated with a blend of 5% hydrogen by volume with natural gas, it consumes around 840 gallons of water per hour to generate hydrogen [64]. Electrolysis also requires electrical power to split apart the water molecules. The required power for an electrolyzer can be obtained by dividing the higher heating value (HHV) by the system efficiency, HHV/η. The HHV for hydrogen is 39.39 kWh/kg. A commercially available electrolyzer with 65% efficiency usually requires 60.61 kWh/kg of water for hydrogen production. The hydrogen flow rate is roughly 11,700 m^3^/h in a GE-10 gas turbine. The electrolyzer system would consume ~1.54 GWh of electricity to generate enough H_2_ to operate the GE-10 for 24 h [76,77].

### 6.5. Recent Research on Hydrogen Fuel-Based Engines

The aeroderivative gas turbine (LM2500+) introduced by GE has been able to utilize coke oven gas (COG) as fuel for power generation. COG has a very high hydrogen content up to 65%. The first two GE aeroderivative COG units could generate 60 MW in a combined cycle configuration.

GE is a well-known manufacturer of gas turbines, which includes various types such as H-class, F-class, B-class, and E-class, and aeroderivative turbines. The HA and F-class turbines are especially renowned for their superior fuel flexibility and high-power output capacity. GE has created a powerful 384 MW 7HA.02 combustion turbine that can burn up to 20% hydrogen by volume while still maintaining a high efficiency and power output. This turbine is also capable of using different gas blends. In addition, GE has been working on developing a multitube combustion system known as the DLN 2.6e [76,77].

Several gas turbine manufacturers are developing technologies to incorporate hydrogen into their products. OPRA, a Dutch turbine OEM, has created a gas turbine called the OP16, which is an all-radial gas turbine and provides high reliability, robustness, and efficiency while lowering the emissions. OPRA has also developed combustor technology that enables its turbines to operate using up to 100% hydrogen. Mitsubishi Power has developed gas turbines that can run on a mixture of 30% hydrogen and 70% natural gas and is working on a turbine that can run on 100% hydrogen. Siemens Energy’s larger gas turbines (from the SGT5-2000E to SGT5/6-9000HL) can operate on up to 30% hydrogen by volume, with plans to develop turbines capable of utilizing higher concentrations of hydrogen in the mid- and long-term future. These gas turbines are used in a variety of industries, including industrial, oil, and gas, and waste-to-energy applications [76,77,78]. 

Kawasaki has successfully demonstrated that their 1 MW M1A-17 gas turbine can operate using 100% hydrogen. The turbine was used to generate electricity and steam for a large event facility and hospital. To address the limited availability of hydrogen, Capstone Turbines has developed microturbines that can be easily installed at hydrogen production sites without the need for additional infrastructure. These turbines serve as distributed energy sources. Capstone recently sold its first hydrogen C65 turbine to a customer in Australia [79].

### 6.6. Hydrogen Fuel Usage in Transportation: Challenges and Limitations

Fuel cells that use a polymer electrolyte membrane can be categorized as either alkaline fuel cells (AFCs) or alkaline membrane fuel cells (AMFCs), which use an alkaline membrane, or proton exchange membrane fuel cells (PEMFCs), which use an acid membrane. While AFCs can employ nonprecious metal catalysts at both the anode and cathode, making them less expensive than PEMFCs, PEMFCs operate at lower temperatures (below 120 °C) and use fluorosulfonic acid as a charge carrier, making them ideal for transportation applications, as they have faster start-up times compared to other types of fuel cells [80].

Alkaline membrane fuel cells (AMFCs) still face challenges, including issues with carbon dioxide tolerance, membrane durability and conductivity, high-temperature operation, power density, water management, and anode electrocatalysis. To improve AMFCs’ performance and efficiency, these limitations must be addressed. Proton exchange membrane fuel cells (PEMFCs) require a significant number of precious metals, primarily platinum, for their construction, which can be costly and limit their widespread use. Therefore, minimizing or eliminating the precious metal use is a research focus for improving PEMFCs’ durability and managing water transport within the cell. Flooding is a common issue in PEMFCs that occurs due to a gas humidity increase, leading to accelerated platinum dissolution–precipitation and carbon support corrosion. The optimization of the flow rates, gas diffusion layer design, and the use of hydrophobic materials are being explored to improve the water management system within the cell, addressing the flooding issue [81,82].

### 6.7. Fuel Cell System: Challenges of Thermodynamic Constraints

Internal combustion engines still cannot perform that well in extreme weather conditions, like subfreezing temperatures. That makes the fuel cell system nondurable for vehicles. Fuel cells can face issues with cold temperatures, as the water contained within them can freeze, reducing their performance until they reach an optimal operating temperature. While fuel cell vehicles (FCVs) can now start and operate in subfreezing temperatures, there are still concerns over their performance in such conditions. Contaminants can also affect fuel cell performance and durability, making it uncertain what level of hydrogen and intake air purity will be necessary for FCVs to operate reliably in real-world conditions. Research is ongoing to address this concern and ensure the reliable operation of FCVs [83]. The other problems of fuel cells include load cycling, which leads to the degradation of the lifetime of fuel cells by developing issues with water management and the dynamic response. Water management affects the catalyst that results in membrane degradation and flooding. Failures like tearing or cracking along with chemical degradation of PEM fuel cells can be accelerated due to the mechanical stress in the membrane caused by dehydration [83,84,85].

## 7. Hydrogen Sensors

Sensors used for detecting hydrogen in the air need to be highly sensitive and responsive, capable of detecting even the smallest amount of hydrogen quickly enough to prevent fires caused by leaks. In particular, the response time of these sensors is critical, with the US Department of Energy setting a target of t90 <1 s at room temperature for detecting hydrogen concentrations in the range of 0.1% to 10%. This target has been elusive since 2007, and the sensors must be able to detect as little as 0.1% hydrogen in the air to be effective. Achieving this level of sensitivity is crucial for ensuring the safety of hydrogen fuel cell systems in both transportation and stationary applications. The major types of sensors for hydrogen parameter measurement are electrochemical sensors, metal oxide sensors, catalytic gas sensors (Cgs), thermal conductivity sensors, optical sensors, palladium film and palladium alloy films, and combined technology sensors [86]. Detecting hydrogen leaks for a robust and safe delivery infrastructure is required from a regulatory body. The current NG sensors are not capable of monitoring leak detection issues caused by hydrogen’s chemical nature. Research efforts have been started on fiber optic sensors for time-dependent infrastructure monitoring and defect detection. Also, refueling sites, stationary storage, and any enclosed areas where hydrogen may be stored are all candidates for hydrogen detection sensors. To prevent mechanical failures and losing stored hydrogen due to leakages, an accurate smart alternative detection sensor should be developed and tested before starting investments in costly infrastructures. Department of Energy (DOE) has fixed a set of specifications to approve hydrogen safety sensor which is summarized at Table 5 [87,88,89].

Hydrogen leaks from any facility, storage system, vehicle, or pipeline should be detected immediately. Optical nano-plasmonic hydrogen sensors based on hydride-forming metal nanoparticles have been introduced. Optical nano-plasmonic hydrogen sensors are a type of sensor that uses hydride-forming metal nanoparticles, such as palladium (Pd), to detect hydrogen gas. These sensors generate optical signals that are highly selective toward hydrogen and produce no sparks, making them safe to use. Pd is an efficient material for hydrogen sensors, because it can dissociate hydrogen gas easily and undergo a reversible phase transformation from metal-to-metal hydride at room temperature. Recently, there has been an increasing interest in using plasmonic metal–polymer coatings, such as Pd or Pd70Au30 coated with polytetrafluoroethylene (PTFE), to improve the sensors’ chemical resistance and hydrophobicity, making them suitable for harsh environments [91,92]. 

Researchers at Chalmers University of Technology in Sweden have developed a hydrogen sensor that can meet the performance targets required for use in hydrogen-powered vehicles. This sensor uses metal nanoparticles to detect hydrogen gas based on an optical phenomenon called a plasmon, which occurs when light is absorbed by the nanoparticles. The sensor is highly efficient and can detect hydrogen gas at a concentration of 0.1% in less than one second, making it the fastest hydrogen sensor developed so far. By meeting the strict performance targets set by the automotive industry, this hydrogen sensor has the potential to play a critical role in the development of safe and efficient hydrogen fuel cell vehicles in the future [92]. H2scan LLC is commercializing hydrogen-specific sensing systems using solid-state technology developed at Sandia National Laboratory. The sensing systems are designed to detect hydrogen gas even in the presence of other background gases. The hydrogen-sensing devices are capable of detecting hydrogen in just 1 to 10 s, which makes them useful for applications that require quick response times, such as control systems. H2scan currently offers three hydrogen-sensing system configurations: a handheld portable leak detector, a fixed-area monitoring system, and an inline real-time concentration analyzer [93]. These sensors have a low hydrogen sensitivity of around 5 ppm in air and less than 1 ppm in nitrogen, making them highly sensitive to hydrogen gas. They are hydrogen-specific, meaning that they are capable of detecting hydrogen without cross-sensitivity to other gases. The upper range of the sensor is 100%, and it has an incredibly fast response time. Furthermore, the sensors can operate within a broad temperature range of −40 °C to 150 °C, making them suitable for a wide range of sensor applications. These features make these sensors potentially useful in many industries, including automotive and chemical manufacturing, where hydrogen gas is commonly used [93].

### Challenges in Developing an Errorless and Economical Hydrogen Sensor

Hydrogen sensing is still a challenge for the hydrogen implications. A combustible gas sensor (hydrogen sensor) has the tendency toward false alarms for other gas presences in the system. False alarms for the other combustible gases make it difficult to use where interference may happen. Premature sensor failure is a common complaint about hydrogen sensors [88,94,95]. Palladium thin films are widely being used for hydrogen sensors. External factors, including temperature, pressure, and relative humidity, have a strong effect on a sensor’s background signal and accelerate degradation. Chemical stressors (contaminants) affect catalyst functionality over time, which leads to sensor failure.

The effectiveness and installation cost are yet to be improved. The sensor certification performance is also subjected to further evaluation and establishment. The positioning of a sensor is critical to optimize the effectiveness. Currently, there is no formal updated guideline for sensor placement from the National Fire Protection Association (NFPA) or the International Fire Code (IFC) [96,97]. Apart from the manufacturing, the regular maintenance of a hydrogen sensor is also still costly. The calibration process with hydrogen test gas is also still a challenge. A low-cost plug is being used as a substitution for performing routine calibrations [88,89]. Hydrogen sensors face three primary challenges: response time, sensitivity, and cost. Currently, the mainstream technology for hydrogen optical sensors involves using a monochromator, which is expensive and time-consuming. The monochromator is used to record a spectrum, which is then analyzed to identify any spectral shift. However, this process can be difficult to achieve high levels of sensitivity in, which is crucial for accurate hydrogen detection. Additionally, the high cost associated with the technology limits its practical application in many industries. All the metals have a tendency to absorb hydrogen. But research is still required for selecting a suitable alloy like palladium cobalt alloy, which can detect the trace changes through light transmission upon absorbing hydrogen. 

One major limitation of hydrogen sensors is their tendency to exhibit hysteretic behavior and inadequate response times that can fall short of the desired target values. The effectiveness of hydrogen dissociation on Pd can also be negatively impacted by trace amounts of other species like CO and NO_2_, which can limit the sensor’s accuracy. However, plasmonic metal–polymer optical hydrogen sensors can help overcome these limitations. By using Pd-Au alloy plasmonic nanoparticles as signal transducers and combining them with specialized thin polymer membrane layers, these sensors can achieve greater accuracy and faster response times. This approach leverages synergistic effects to improve the performance of hydrogen sensors, addressing some of the key challenges associated with traditional hydrogen sensing technologies [98,99,100,101,102].

Hydrogen is odorless but highly volatile and flammable. Only 4% hydrogen in the air is capable of igniting at the smallest spark. Therefore, highly efficient hydrogen sensors are obvious for hydrogen cars and the associated infrastructure in the electricity network industry, the chemical industry, and the nuclear power industry. 

## 8. Hydrogen Safety

The safety and reliability of the required infrastructure are necessary conditions for the hydrogen economy (HE) to become a reality. The system designs should be robust, with the ability to demonstrate levels of safety equivalent to, or safer than, the currently used technology.

The safety and reliability of hydrogen-induced infrastructures are the most vital concerns to build up the hydrogen economy. The infrastructure includes hydrogen production, storage, and transportation facilities. Steam reforming is one of the most popular hydrogen production technologies. But the steam methane reforming unit has the presence of flammable conditions at the desulphurization unit, which causes a high fatality rate. The lowest fatality rate is observed by the hydrogen purification absorber. The maximum radiation by jet fire is also caused by a full-bore rupture in a desulphurization reactor in both the summer and winter seasons. Extensive studies are yet to be done to assess and mitigate the risk involved in the hydrogen production system.

Liner blistering in a pressure vessel is an associated risk for hydrogen storage and transport. Plastic liners absorb hydrogen gas, and the accumulated gas cannot be released if depressurization takes place rapidly. Pepin et al. [103] built a test rig that enabled replicating liner blistering and separation on small samples by explosive decompression rather than performing tests on cylinders to understand the mechanism of the liner failure. Wu et al. [104] also studied the damage mechanism of carbon fiber through experimental and numerical analyses by varying the duration of the impact and magnitude of the impact force.

Resistance to fire and high temperatures of a hydrogen carrier are significantly important factors at different operating conditions. Ruban et al. [105] did the bonfire test on fully composite hydrogen storage vessels and showed that the increase in pressure before bursting or leakage is minor (maximum 12.7%), and the burst delay (time before burst) is in the range of 6–12 min, depending on the initial pressure of the vessel, which is not acceptable.

Hydrogen leakage is another concern for hydrogen-assisted infrastructures due to the nature of hydrogen molecules. It results in leak rates of hydrogen through steel and seals three times greater than natural gas. An analysis performed on Germany’s natural gas pipeline showed a 0.00005% gas leakage rate for a 17% hydrogen and natural gas blend. Further research and empirical data are needed to obtain a better gas loss estimate.

Pressure fluctuations in pipelines may severely damage the distribution network resulting from the demand variability of hydrogen gas. Yu et al. [106] studied different loadings by applying them on X60 steel pipes, and it was shown that pressure cycles can accelerate the corrosion crack propagation by a factor of 2.7 and 5.3 for tests in air and near-neutral conditions. These results are alarming enough to motivate further detailed analyses on this topic.

## 9. Hydrogen Storage and Transport: Research and Opportunity

In addition to pipeline systems, hydrogen, as well as other fuels, are transported on ground pressure vessels and barges. Tube trailers with up to 800 kg capacity at a pressure of 250 bar are commonly used to distribute hydrogen gas within 320 km of the source. Hydrogen is also economically distributed through hydrogen tanker trucks with capacities of 4–5 tons and a reach of 950 km. These types of transport vehicles are currently regulated in the United States by the Department of Transportation (DOT), which allows pressures up to 250 bar. However, special permits have been granted for hydrogen transport at a pressure of 500 bar [107].

One of the main challenges with the storage and transport of hydrogen are the losses of hydrogen fuel due to boil-off. Hydrogen leaks can take place at both the liquid state and gaseous state. Commercially available leak detection equipment has a short detection range and thus is handheld. An inline detection system would be a desirable improvement in the monitoring and assessment of losses from transport vehicles [107].

Similarly, the development of advanced insulation technologies and high-capacity liquid organic carriers (LOHCs) may also be able to allow for longer travel distances at high capacity [108]. However, some of the technical disadvantages need to be addressed with LOHCs. Likewise for the high pressure and temperature required for the hydrogenation and hydrogen release processes, respectively, along with the high quantity and cost required. Apart from that, poisoning of the catalyst during dehydrogenation and formation of the intermediate process during hydrogen release are also important. The reversibility of hydrogenation and dehydrogenation, hydrogen capacity, and carrier cost are also important factors to be addressed for LOHCs.

Tanks storing compressed gaseous hydrogen or liquid hydrogen have high discharge rates and efficiencies that make them appropriate for small-scale applications where a stock of fuel needs to be readily available. However, compressed gaseous hydrogen has an energy density 15% that of gasoline [109]. This aspect of hydrogen as an energy source means that storing equivalent amounts of energy would require nearly seven times the space compared to hydrocarbon fuels. Further research is needed to reduce the size of the storage vessels. This would also include increasing the scope of the underground storage pressure to 800 bar for large-scale storage [1,8,110,111]. The vessels used for the storage of this gas are shown in Table 6.

From the summarized types of storage vessels, type 3 and 4 are the most common in industrial uses. Elevated pressure is the key difference between the storage of hydrogen and storage of other fuels [112]. In the case of high-pressure refueling stations, hydrogen is typically stored in thick metallic liner hoop vessels with fiber–resin composite coatings. This design has been identified as one of the major contributors in the elevated price of hydrogen delivery infrastructures. For this reason, further research is required to understand the effects of high-pressure charge and discharge cycles, as well as the environmental effects on the integrity of the pressure vessels employed [113].

Similarly, liquid hydrogen tanks are currently used to store large quantities of hydrogen, particularly at fueling stations, because liquid storage provides a much higher volumetric efficiency than gas storage. Stations that use liquid hydrogen convert it to high-pressure gas through a process of pumping, vaporization, and compression prior to dispensation. This cryogenic storage method is typically sized to satisfy the demand for 7–10 days. Nevertheless, one of the main disadvantages of liquid storage is the need for temperatures close to or lower than 20 K (−253 °C) to sustain the liquid phase. Furthermore, regardless of the equipment design, it is not possible to fully mitigate hydrogen boil-off. For these reasons, the development of improved cryogenic storage systems is required to achieve pressure containment at low temperatures, as well as better insulation materials [8].

In contrast, an innovative concept that aims to reduce storage costs and increase the volumetric efficiency of hydrogen as an energy source is the use of solid carriers within a lower-cost storage tank. Through the use of a metal hydride or a specialized nanostructure, it would be possible to store hydrogen gas molecules in lower-pressure tanks. Nevertheless, these kinds of storage systems require low temperatures to absorb hydrogen and heat to release the gas molecules. Research on these processes is needed in order to optimize the thermodynamic exchange of the carrier system to maintain low costs [8].

In the case of large-scale hydrogen storage, underground features such as mines, salt caves, oil and gas reservoirs, and aquifers are routinely used to provide storage during seasonal increases in natural gas production. A large-scale hydrogen storage infrastructure would require some of the same volume characteristics. Currently, there are five locations that offer large-scale underground hydrogen storage: four salt caverns in the state of Texas and one cavern in Teesside, England. Nevertheless, development costs, contamination concerns, natural losses, and geographic limitations remain to be addressed [114].

In a similar way to other large storage vessels, the cushion gas that remains in the geological feature represents a significant cost. Experience with natural gas storage suggests that a cushion would amount to 15% of the storage capacity. Furthermore, there is an incomplete understanding of the effects of pressure cycling on rock formations. This rock mass used as the storage vessel might not be a continuous medium, which could create unexpected formations, as well as undesired chemical reactions [110,115].

Salt caverns, depleted natural gas and oil reservoirs, and aquifers are possible options. Salt caverns have been used for hydrogen storage by the chemical sector of the United Kingdom and the United States since the 1970s. Their high pressures enable high discharge rates, which makes them attractive for the industrial and utility sectors. Since salt caverns are typically operated as a series of separate adjacent caverns, it would be possible to adapt the technology into using natural gas storage facilities, reducing the initial costs [8].

Depleted oil and gas reservoirs are typically larger than salt caverns. However, they also tend to be more permeable and contain pollutants that would have to be evacuated prior to hydrogen storage. Similarly, aquifers are the least-studied geological storage option due to concerns over sustainability of the practice. In both of these geological features, microorganisms, fluids, and minerals could react with the stored hydrogen, causing losses and contamination. Although geological storage presents itself as a beneficial option for long-term and large-scale hydrogen storage, their large size and minimum pressure characteristics would make them less suitable for short-term economical storage [116,117].

## 10. Conclusions

The idea of using hydrogen as a fuel source in order to reduce greenhouse gas emissions is an ambitious and altruistic notion. Research is being done all across the United States and the world to find inexpensive and safe ways to produce hydrogen to proliferate the dream of a hydrogen economy. Reducing greenhouse emissions (GHGs) is the key driving force behind concentrating on hydrogen for use as fuel. Research has been carried out globally, with the top priority to find a feasible route for establishing a hydrogen economy through satisfying the technical and commercial challenges. This article provides an overview of hydrogen compatibility and suitability for applications in various aspects in terms of research potentiality, which will help to understand the industry leaders and research groups for future focus on a hydrogen world. The US Department of Energy (DOE) aims to accelerate breakthroughs of more abundant, affordable, and reliable clean energy solutions within the decade. Currently, the bulk applications of hydrogen are limited to chemical industries like oil refining, methanol production, and ammonia production and the manufacturing industry like steel production. In various aspects, the global economy and human lives have been bolstered by these existing uses of hydrogen. Presently, hydrogen production is mostly dependent on natural gas, coal, oil or fossil fuel, which comes with severe environmental impacts. However, there are technologies available to avoid emissions from fossil fuel by producing and supplying low-carbon hydrogen. Associated challenges have been discussed for environmentally friendly and economical hydrogen uses in the industries in this article.

Our thorough analysis provides important suggestions to improve hydrogen technology. These include using better materials for storage and transmission, finding ways to prevent hydrogen embrittlement, improving how we make hydrogen, creating new storage solutions, refining fuel cells, developing a complete hydrogen system, checking the environmental impact at every stage, making strong safety rules, finding ways to make hydrogen more affordable, and telling the public more about hydrogen. Following these ideas can help us make progress, solve problems, and create a better future with hydrogen.

Addressing the challenges highlighted in hydrogen technology is of paramount importance in shaping its future as a green energy source. Overcoming these challenges can yield transformative outcomes on multiple fronts. Enhancing the materials for transmission and storage, coupled with strategies to mitigate embrittlement, extends the lifespan of hydrogen infrastructures, ensuring safety and reliability. Advancing hydrogen production methods and CO_2_ capture technologies contribute to a more sustainable energy cycle, minimizing environmental impacts. Additionally, surmounting challenges spurs innovation, fostering economic growth and job creation. This progress aligns with the climate change mitigation goals by enabling hydrogen’s role in decarbonizing challenging sectors. Moreover, overcoming challenges creates a self-reinforcing cycle of technological advancement, driving further investments and scalability. Ultimately, these efforts align with the global sustainability objectives, offering an alternative to fossil fuels and enhancing energy security. In conclusion, addressing hydrogen challenges is pivotal for realizing its potential as a sustainable and transformative energy solution with widespread positive impacts.

## Figures and Tables

**Table 1:** Sources and methods of gaining different types of hydrogen [12,13,14].

H_2_ Type	Source of H_2_	Method	Emission Type
Green H_2_	Renewable/Nuclear and Biomass	Electrolysis	Oxygen
Pink H_2_	Nuclear energy	Electrolysis	Nuclear Waste
Pure H_2_	Electricity from Grid	Electrolysis	
Gray H_2_	Natural gas	SMR	CO_2_
Blue H_2_	Coal	Gasification (With CCUS)	CO_2_
Brown H_2_	Coal	Gasification (Without CCUS)	CO_2_
Black H_2_	Coal	Gasification (Without CCUS)	CO_2_

**Table 2:** Summary of the challenges and opportunities of selective hydrogen production methods [12,13,14,33,34].

Methods	Critical Challenges	Major R&D Needs	Opportunities	Present Status
SMR	High capital costs	Cost effective process	Sustainable approach	Advanced process
	High operation and maintenance costs	Efficient purification techniques with low cost	Lowest current cost	Application in fuel cell electric vehicles (FCEVs)
	Design issues	Optimization and Reliability	Existing infrastructure	
	Feedstock management	Feedstock pretreatment	Produced CO_2_ can be used as byproduct.	
		Automated process control	CO_2_ emission can be reduced if combined with CCUS	
Electro-lysis	High capital costs	Durable and cheap materials	No pollution with renewable energy sources	Alkaline electrolysis is mature and commercial technology
	Low system efficiency	Corrosive-resistant membranes	Existing infrastructure	SOECs yet to be developed and commercialized
	System integration	Durable, active, and cheap catalysts	Integration with fuel cells	Application in Fertilizer and Chlorine industry
	Access of freshwater	Large scale applications and Reliability	Thinner membrane with high mechanical strength can increase cell efficiency	
	Expensive catalyst	Storage and production rate	Integration of heat from various sources	
Coal Gasification	High reactor costs	Efficient purification techniques	Production of syngas with low cost	Production of Ammonia in Chemical and Fertilizer industry
	Feedstock impurities	Co-fed gasifiers	Abundant and cheap feedstock	Mature Technology
	Carbon capture and storage	Carbon capture and storage technology	Reduce the volatilization of coal can increase efficiency	New membrane technology for H_2_ separation and purification
	High CO_2_ emissions intensity	Hydrogen quality	CO_2_ can be decarbonized if combined with CCUS	
	lack of established standard codes	Feedstock preparation cost		
	Cool the hot gas above 1400 °C	Tolerance for impurities		.
Biomass Gasification	High capital costs	Low cost and efficient purification	Production of syngas with low cost	Mature Technology
	Feedstock impurities	Co-fed gasifiers	Abundant and cheap feedstock	New membrane technology for H_2_ separation and purification
	Carbon capture and storage	Carbon capture and storage		Commercial demonstration
		Hydrogen quality		
		Cost of feedstock preparation		
		Tolerance for impurities		

**Table 3:** A comparative review of the hydrogen storage capacities of different hydrogen storage materials [58,59,60,61,62,63,64].

Storage Method	Storage Materials	Storage Capacity, (wt.%)	Pressure (bar)	Temperature (K)
Compressed Gas	Energy for compression: ≈4 kcal·mol^−1^	13	140	298
Cryogenic Liquid	Energy for liquefaction: ≈7 kcal·mol^−1^	Depending on size (e.g., ≈5 wt.% for a can tank	1	21
Adsorption	Activated Carbon	5.5	80	298
	Graphite	4.48	100	298
	Single-walled carbon nanotube	4.5	4	298
	Multiple-walled carbon nanotube	6.3	148	300
	Carbon nanofiber (CNF)	6.5	120	300
Absorption	AB_5_ type: LaNi_5_	1.37	2	298
	AB_2_ type: ZrMn_2_	1.77	0.001	298
	A_2_B type: Mg_2_Ni	3.59	1	555
	AB type: FeTi	1.89	5	303
	NaAl	5.6		493
	LiAl	7.9		453

**Table 4:** Hydrogen embrittlement susceptibility of commonly used metals adopted from [66,67].

Metal	Extremely Embrittled	Severely Embrittled	Slightly Embrittled	Negligible Embrittled
Aluminum alloys				
1100				Yes
6061-T6				Yes
7075-T73				Yes
Be-Cu alloy 25			Yes	
Copper, OFHC				Yes
Nickel 270		Yes		
Titanium and titanium alloys				
Titanium			Yes	
Ti-5AI-2.5SN (ELI)		Yes		
Ti-6AI-4V (annealed)		Yes		
Ti-6AI-4V (STA)		Yes		
Steel				
Alloy steel, 4140	Yes			
Carbon steel				
1020		Yes		
1042 (normalized)		Yes		
1042 (quenched and tempered)	Yes			
Maraging steel, 18Ni-250	Yes			
X42			Yes	
X52			Yes	
X60			Yes	
X65			Yes	
X70			Yes	
X80			Yes	
X100			Yes	
Stainless steel				
A286				Yes
17-7PH	Yes			
304 ELC			Yes	
305			Yes	
310				Yes
316				Yes
410	Yes			
440C	Yes			
Inconel 718	Yes			

**Table 5:** DOE-targeted specifications for hydrogen safety sensors R&D [87,88,89,90].

Parameter	Value
Range of Measurement	0.1–10%
Operation Temperature	−30–80 °C
Minimum Response Time	<1 s
Accuracy and Precision	5% of full scale
Gas environment	Ambient air, 10% to 98% RH
Average Lifetime	10 years

**Table 6:** Vessel types used for the storage of gas [112].

Type I	Metal pressure vessel
Type II	Thick metallic liner hoop with fiber–resin composite coating pressure vessel
Type III	Thin metallic liner with fiber–resin composite coating pressure vessel
Type IV	Polymer liner with fiber–resin composite pressure vessel

## Data Availability

Not applicable.
